# People underestimate the errors made by algorithms for credit scoring and recidivism prediction but accept even fewer errors

**DOI:** 10.1038/s41598-021-99802-y

**Published:** 2021-10-11

**Authors:** Felix G. Rebitschek, Gerd Gigerenzer, Gert G. Wagner

**Affiliations:** 1 Harding Center for Risk Literacy, Faculty of Health Sciences Brandenburg, University of Potsdam, Potsdam, Germany; 2 Max Planck Institute for Human Development, Berlin, Germany; 3 German Socio-Economic Panel Study (SOEP), Berlin, Germany

**Keywords:** Human behaviour, Information technology

## Abstract

This study provides the first representative analysis of error estimations and willingness to accept errors in a Western country (Germany) with regards to algorithmic decision-making systems (ADM). We examine people’s expectations about the accuracy of algorithms that predict credit default, recidivism of an offender, suitability of a job applicant, and health behavior. Also, we ask whether expectations about algorithm errors vary between these domains and how they differ from expectations about errors made by human experts. In a nationwide representative study (N = 3086) we find that most respondents underestimated the actual errors made by algorithms and are willing to accept even fewer errors than estimated. Error estimates and error acceptance did not differ consistently for predictions made by algorithms or human experts, but people’s living conditions (e.g. unemployment, household income) affected domain-specific acceptance (job suitability, credit defaulting) of misses and false alarms. We conclude that people have unwarranted expectations about the performance of ADM systems and evaluate errors in terms of potential personal consequences. Given the general public’s low willingness to accept errors, we further conclude that acceptance of ADM appears to be conditional to strict accuracy requirements.

## Introduction

This study provides the first representative analysis of error estimations and willingness to accept errors in a Western population (in Germany) with regards to specific algorithmic decision-making (ADM) systems. We examine how accurately algorithms are expected to perform in predicting credit defaults, recidivism of an offender, suitability of a job applicant, and health behavior.

Algorithmic decision-making (ADM^1^) continues to spread into everyday life. At the same time, claims, risks^2–4^, and implementations related to ADM are under debate, e.g. in criminal risk assessment^5–7^ or allocation of public resources^8^. Despite these controversies, it is unclear how the general public perceives ADM quality. Representative studies focus on attitudes^9–11^ and on perceived understanding and application of ADM and artificial intelligence^12–15^, but rarely on perceptions of the systems’ reliability and validity (for an exception, see^16^). The public debate centers on laypeople’s trust in and fear of algorithms^16^, e.g. in media coverage of hopes and concerns regarding artificial intelligence^17^. Research on algorithm aversion and appreciation^1,18^ examines both the circumstances under which people trust algorithmic advice^18,19^—for instance, because they perceive the decision problem in question to be objective or to require mechanical skills^20^ or because they lack confidence in their own expertise—and the circumstances under which they are mistrustful, for instance^21,22^, in response to slow algorithm responses or to observing algorithm errors.

Layperson’s knowledge about ADM systems is usually limited^12^, if the algorithms themselves are not even secret. This is where layperson’s theories about people's ADM systems—their theory of machine—become crucial^18^. What do they think about input, processing, and output, and, moreover, about quality and fairness of algorithmic compared to expert judgments? Given the limited possibilities to actually observe ADM errors, expected instead of observed performance deficits may underpin critical attitudes of the public toward ADM. Extremely high performance expectations, for instance, could underlie algorithm aversion, when they provide a mental reference point that is failed by an algorithm^23^. But what are the people’s expectations with regard to ADM performance?

Surprisingly, the expected level of accuracy of algorithms and the perceived competence^24^ of algorithmic advice have been largely neglected in research on the general population. Fifty-eight percent of Americans expect some level of human bias in ADM systems, and 47% and 49% respectively believe that resume screening of job applicants and scoring for parole are “effective”^16^. In the present article, we aim to reduce the gap in knowledge about the general public’s concrete expectations by investigating what they expect regarding the accuracy of ADM in the financial, legal, occupational, and health domains. We ask people what they believe are the actual error rates made by ADM and how many errors they consider to be acceptable and check whether their responses meets current ADM accuracy standards (in the financial and legal domains). To the best of our knowledge (after a literature search in Web of Science, PsycNet, and Google Scholar, which revealed eleven survey studies on algorithm perception^9,10,12–17,25–27^), this is the first representative study comparing error estimates and the willingness to accept errors for ADM.

Classifying ADM systems balance two different types of errors, misses and false alarms, each associated with different consequences (costs). Thinking about types of decision errors and related costs can affect acceptance of errors (e.g. ‘bias’ in signal detection theory^28^; cost-sensitive error management^29^), and the degree to which errors are accepted may differ in the legal^30^ and in the medical domain^31^, e.g. if the consequences of medical errors are irreversible. From the perspective of an unemployed person, for instance, mistakenly being overlooked for a job by an ADM (a false negative) is likely more costly than being hired in spite of being unsuitable (a false positive). In our analysis, we therefore relate error acceptance to critical factors such as unemployment phases. Also, we compare the willingness to accept errors with the typical error preference of exemplary stakeholders (e.g. non-recidivizing offenders want to avoid a false alarm). We additionally explore factors underlying attitudes toward technology, that may influence both algorithm error estimation and acceptance, such as risk preference^32^, gender^12,33^, and age^34^. For instance, only one third of US-Americans above 50 years of age compared with half of those 18 to 29 years of age believe that algorithms can be free of human biases^16^. Because algorithm appreciation was shown to be lower among less numerate people^18^, we compare different educational groups.

Best-selling authors and commercial companies have promoted “AI” as being superior to human experts^35^, and in some instances AI has indeed demonstrated better performance for selective tasks (e.g. image classification^36^ and deceptive text detection^37^). However, results from earlier studies indicate that in the medical domain, people actually prefer human experts over automated care because, among others, they believe that machines disregard individual circumstances^38^. People were also found to trust recommendations of computers less than those made by physicians^39^ and, crucial for error perception, to consider algorithm-based advice as being less accurate than clinicians’ advice^40^. In the domain of people analytics—algorithm-based planning, recruitment, development, and promotion in human resources—human interviewers are also assumed to be more accurate and useful than algorithmic decision aids^41^. At least for those two domains, the few existing studies thus indicate that people perceive algorithms to make more errors than human experts do. Accordingly, we compare error estimation and error acceptance for algorithmic and expert advice across domains.

We conducted a survey of a large population-representative sample of members of private households in Germany (by means of the Innovation Sample of the German Socio-Economic Panel Study, SOEP IS). In the survey, 3086 respondents were questioned about estimated and accepted error rates in current credit scoring before being randomly assigned to decision scenarios either on algorithms or on experts (between-subjects). Respondents were requested to provide estimated and accepted error rates in a people analytics problem (predicting a suitable job candidate), a legal problem (predicting recidivism of an offender), and a health problem (evaluating health behavior). For instance, respondents were asked with respect to credit scoring: “You have probably heard of the Schufa. The Schufa estimates the solvency of all persons in Germany. This helps entrepreneurs and landlords decide whether someone gets a cell phone contract, an apartment, or a loan, for example. Now please imagine a group of 100 people who are actually insolvent: How many of them do you think are mistakenly assessed by the Schufa as being solvent? [estimating false negatives]; What would be acceptable to you: At most, how many of these 100 people could be mistakenly assessed by the Schufa as being solvent? [accepting false negatives].

## Results

### Estimated and accepted error rates of ADM systems

**Figure 1:**
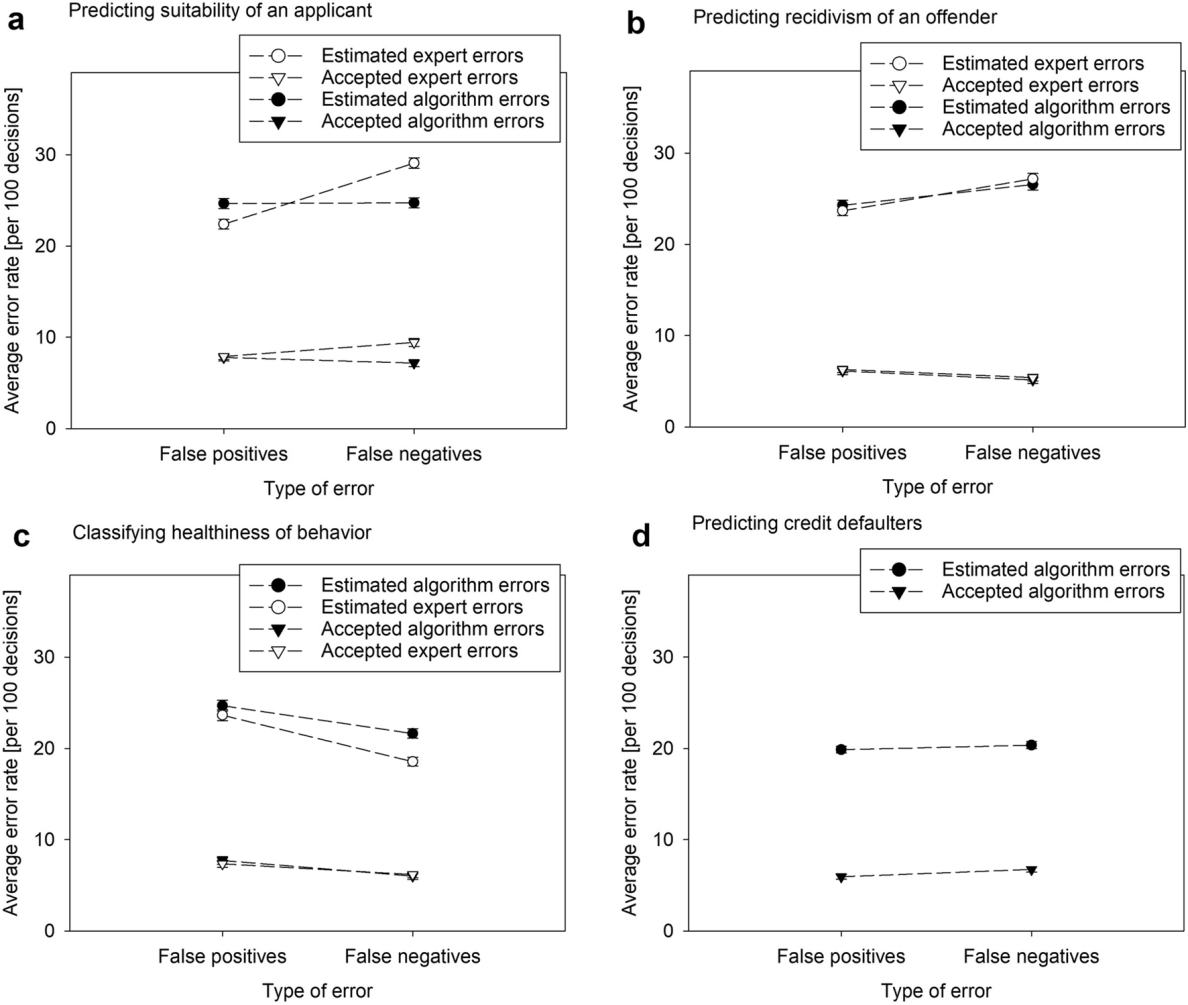
**(a–d)** Estimated and accepted rates of false positives and false negatives related to the assessments of job suitability (**a**), recidivism (**b**), and health behavior (**c**) by algorithms and experts, and to credit scoring (**d**). Data are weighted for representativeness. Error bars show the standard errors of the mean. For predicting credit defaulters, estimates of expert errors were not elicited.

Across domains and types of errors, about one in every four algorithmic decisions was estimated to be wrong (Fig. 1a–d), with the lowest estimates of errors for credit scoring (Fig. 1d). Only a few respondents believed in perfect algorithm (or expert) performance (Supplementary Table S1).

Minimally more false negatives than false positives were estimated for the algorithmic prediction of recidivism (*F*(1, 1314) = 15.75, *P* < 0.001, *η*_p_^2^ = 0.01), but false negatives were considered less acceptable (*F*(1, 1321) = 4.32, *P* = 0.038, *η*_p_^2^ < 0.01). For the algorithmic evaluation of health behavior (Fig. 1c), more false positives (falsely healthy) than false negatives were estimated (*F*(1, 1297) = 39.17, *P* < 0.001, *η*_p_^2^ = 0.03) and considered acceptable (*F*(1, 1310) = 22.76, *P* < 0.001, *η*_p_^2^ = 0.02).

Notably, there is a consistent and large difference between the estimated and maximally accepted rates of algorithm error across all four domains. Expressed in percentage points, the differences in false positives (FP) and false negatives (FN) are for application suitability (Δ_FP_ = 17, Δ_FN_ = 17), for recidivism (Δ_FP_ = 18, Δ_FN_ = 21), for health behavior (Δ_FP_ = 17, Δ_FN_ = 16), and for credit scoring (Δ_FP_ = 14, Δ_FN_ = 14).

### Estimated and actual errors of ADM systems

**Figure 2:**
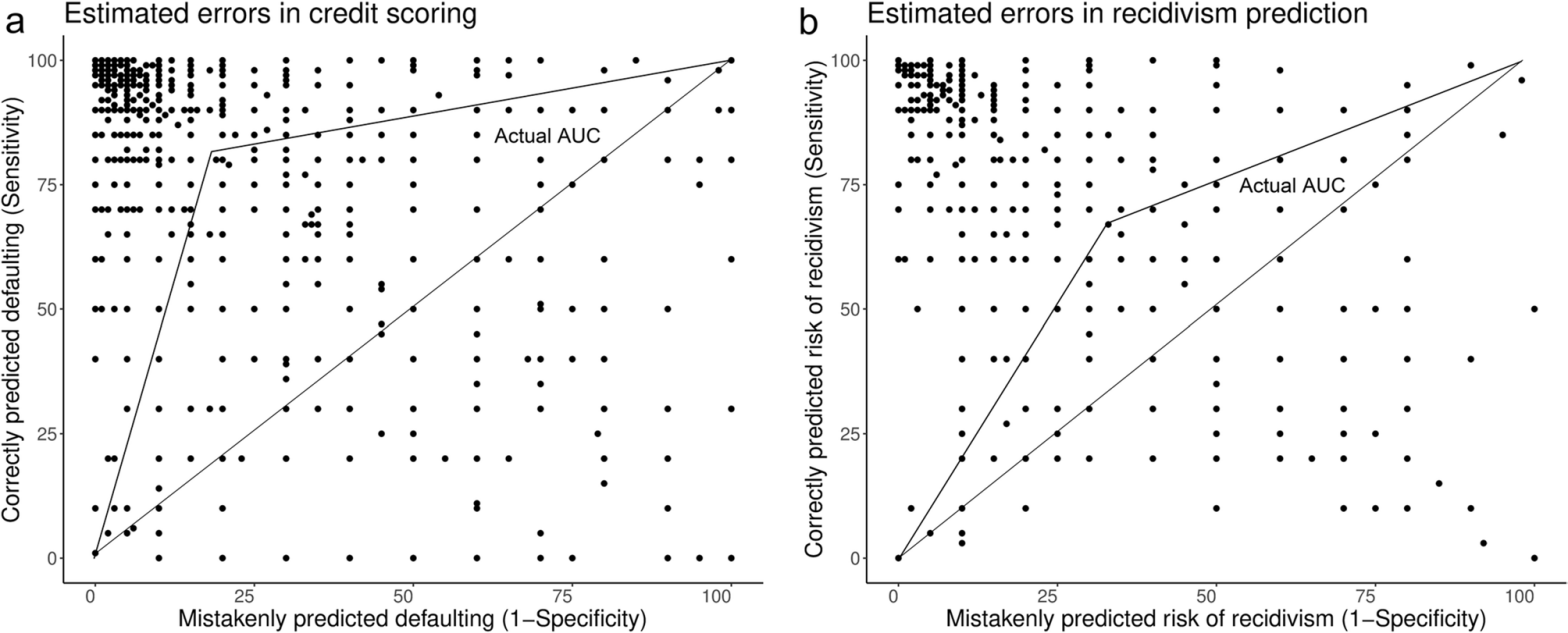
**(a,b)** Respondents overestimate the accuracy of algorithms for credit scoring and recidivism prediction. The scatterplots show each respondent’s estimates of the false positive rate (1—specificity) and false negative rate (shown as its complement, the sensitivity) in credit scoring (*n* = 2740) and in recidivism prediction (*n* = 1325). Each point corresponds to one respondent. The triangles frame the areas under the simplified ROC curves for actual credit scoring accuracy (SCHUFA Credit Score algorithm, AUC = 0.81) and for recidivism prediction (COMPAS algorithm, AUC = 0.68).

To assess whether estimated algorithm errors correspond to actual errors, we compared respondents’ estimates with actual performance metrics for recidivism and credit scoring algorithms (for the scenarios of health behavior and people analytics, reliable metrics are not publicly available). Figure 2a (left) shows each of the respondents’ estimates for the false negative rate and false positive rate, expressed as sensitivity (1—false negative rate) and 1—specificity (false positive rate). Each dot corresponds to one respondent. Given that the SCHUFA does not provide data for the entire curve, we depict simplified areas under the ROC curves with assumed error balance and linear decrease to the extreme values. The areas are represented by the triangles on the diagonal from bottom left to top right. The dots above the diagonal represent an overestimation of the accuracy of the credit scoring algorithm and the dots below represent an underestimation.

The credit agency SCHUFA reports an accuracy of 0.81 of its (banking) credit score in terms of the value of the area under the receiver operating curve (AUC)^42^. This appears to be similar to the AUC_WeightedMean_ = 0.80 (SD = 0.17) that we calculated (geometrically, assuming a linear performance decrease to the extreme values) based on each respondent’s error estimates. However, 45.5% of participants (95%CI [43.6%, 47.5%]) overestimated AUC (> 0.85), while 33.0% (95%CI [33.1%, 33.5%]) underestimated it (< 0.76). Underestimations were more extreme than overestimations.

Participants who underestimated or overestimated the accuracy of credit scoring algorithms differed in several respects from those who gave approximately correct estimates (Supplementary Table S2). Underestimators had lower formal education, and about 500 euros less household income per month. They were more likely not employed, with phases of unemployment in the past ten years, and they also reported having a worse state of health. Overestimators had more often completed college education than those who estimated correctly. Generally, the youngest participants (17 to 29 years) and those with the lowest net household income (up to 1500€) expected and accepted the highest error rates in credit scoring (Supplementary Fig. S1). The lowest rates were expected and accepted by academics and those from the highest income group (from 4501€).

The same analysis is shown for the prediction of recidivism in Fig. 2b (right). It is important to consider that official statistics underlying recidivism prediction show only data about people who reoffend and are arrested, while those who reoffend and are not caught are missing. Given this constraint, the accuracy of the widely used COMPAS algorithm (General Recidivism Risk Scale, any offence) is lower than that of the credit scoring algorithms, with an AUC of 0.68^43^. This is lower than the AUC_WeightedMean_ = 0.75 (SD = 0.19) that we calculated based on each respondent’s error estimates. Moreover, 61.0% of participants (95%CI [58.3%, 63.7%]) overestimated AUC (> 0.73), while 24.9% (95%CI [22.5%, 27.2%]) underestimated it (< 0.63). Participants who underestimated the accuracy of the recidivism algorithm (Supplementary Table S3) had lower formal education, lower household income (− 300 euros), and more likely phases of unemployment in the past ten years. Overestimators were on average five years younger, more likely reported full time employment, and had completed college education more often than those who estimated correctly.

The performance of the algorithm does not meet the expectations of major parts of the population, as expressed in estimations and acceptance. We characterize this gap and its relevance in the following section. For a characterization of distinct groups with lower and higher estimates and acceptance of algorithms in determining applicant suitability and assessing healthy behavior, see Supplementary Tables S7, S8.

### Acceptance of different errors made by ADM systems

**Figure 3:**
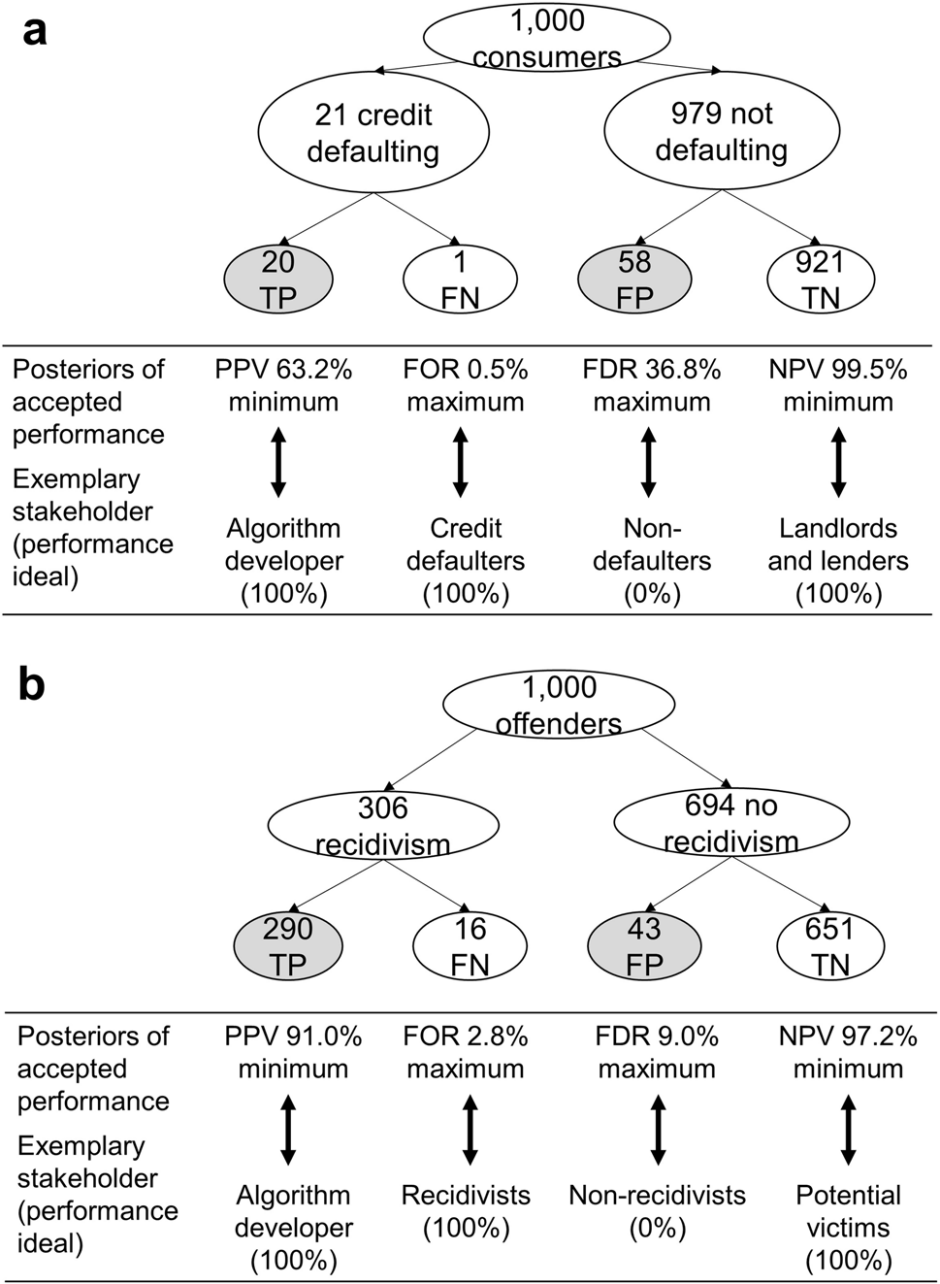
**(a,b)** Natural frequency trees^45^ of algorithm error acceptance for base rates of 2.1% credit defaulters (**a**) and of 30.6% recidivists (**b**), and comparison of minimum performance acceptance (posterior probabilities) of respondents with performance ideals of exemplary stakeholders. Posteriors shown are the acceptable minimum PPV (positive predictive value; e.g. that a person will commit another crime if testing positive), minimum NPV (negative predictive value: e.g. that a person will not commit another crime if testing negative), maximum FOR (false omission rate = 1-NPV), and maximum FDR (1—PPV); TP (true positive), TN (true negative), FP (false positive), FN (false negative). Note that averages of posteriors that were calculated on the individual level are not equal to posteriors based on aggregated TPs, FPs, TNs, and FNs.

To interpret the absolute figures of accepted errors, the base rates of the targets of the algorithms (e.g. how many people default within one year) need to be taken into consideration. What directly counts for a person affected by an algorithm are the posterior probabilities (how likely a certain prediction is correct or incorrect), not the false negative rates (how many of the targets are missed) and false alarm rates (how many of the non-targets are falsely recognized as being a target). In a first step, we calculated implied posterior probabilities with the rates of accepted algorithms errors (weighted for representativeness) and the reported base rates of 30.6% for recidivism (occurring within four years, any offense, both genders; Table 2 in^43^) and of 2.1% for credit defaulting (occurring within one year^44^). Figure 3a shows that for those consumers who do not pay back an agreed credit in time (the 21 out of 1000 in the real world), each respondent accepted a certain proportion of misses, and so required a certain detection rate of true positives (on average 20 out of 21). For the non-defaulting consumers (979 out of 1000), each respondent accepted a certain number of false positives (on average 58 out of 979). The proportion of true positives among all positives is the accepted minimum positive predictive value (PPV, also termed precision: how likely a recognized target is correct). Averaged across PPV calculations on the individual level, the required PPV of credit defaulting is 63%, whereas that for recidivism is about 91%, whereas the credit algorithm actually is more accurate than the one for recidivism.

In a second step, we compared the calculated posterior probabilities of error acceptance with ideal posteriors according to different stakeholders’ goals (Fig. 3b). For algorithm developers, the key goal is to increase the PPV in the direction of 100%^46^. An offender at risk of recidivism, in contrast, wants to falsely test negative and be released, that is, prefers a test with a high false omission rate (FOR). In contrast to offenders at risk, those not at risk have an interest in not being falsely diagnosed as at risk of recidivism (low false discovery rate, FDR). Finally, potential victims of defenders on bail will desire tests that maximize the negative predictive value (NPV) toward 100%, that is, the probability that offenders will not commit another crime if they are tested negative and are released. The comparison showed that people’s preferences for recidivism prediction overlap with performance ideals of developers, of potential victims, and of non-recidivizing offenders, but not with recidivizing offenders: False negatives were considered less acceptable than false positives.

Figure 3a provides similar examples of conflicting interests for predicting default. For instance, landlords and lenders do not want to contract with defaulting consumers; thus, their goal is to reach an NPV that approaches 100%. Customers at risk of default, in contrast, may perceive themselves as profiting from tests that have a low NPV, which is equivalent to high FOR. They may have an interest in being granted credit despite the risk of not repaying it. People’s error acceptance did partially match performance ideals of the developers. Of note, our sample signaled substantial dissent with non-defaulters because many false positives—more than false negatives—were considered acceptable.

Different posteriors lie in different stakeholders’ interest, because they expect personal consequences from different types of errors. Hence, it is examined in the following how error acceptance could depend on personal consequences from errors in specific domains.

### Factors of domain-specific ADM error acceptance

A linear regression (*F*_FP_(6, 540) = 38.38, *P* < 0.001, *R*_corr_^2^ = 0.29) that controlled for error estimations, age, income, and gender showed that respondents who were unemployed within the past ten years (std. *β* = 0.15) showed increased acceptance of falsely assessed suitability by algorithms. Noteworthy, this was not predictive in a parallel analysis of expert assessments (*P* = 0.826). Overlooking suitable job candidates (*F*_FN_(6, 545) = 31.33, *P* < 0.001, *R*_corr_^2^ = 0.26) by algorithms was less accepted by older people (std. *β* = − 0.15). Again a relationship that could be not indicated for experts (*P* = 0.256).

Furthermore, a linear regression (*F*_FN_(5, 820) = 15.41, *P* < 0.001, *R*_corr_^2^ = 0.09) revealed that misses (false negatives) of recidivizing offenders were less accepted by people with higher income (std. *β* = − 0.07), and women (std. *β* = − 0.07). False positives by algorithms (*F*_FP_(5, 809) = 17.08, *P* < 0.001, *R*_corr_^2^ = 0.10) were less accepted with increasing age (std. *β* = − 0.12) and income (std. *β* = − 0.10). We could neither confirm for false negatives (*P* = 0.064 and *P* = 0.378, respectively) nor false positives by experts (*P* = 0.343 and *P* = 0.583, respectively) the listed factors.

If additionally controlling for body mass index, health, and insurance status, a false positive algorithmic evaluation of health behavior (*F*_FP_(8, 782) = 21.43, *P* < 0.001, *R*_corr_^2^ = 0.18)—corresponding to overlooking unhealthy behavior—was less often accepted by older people (std. *β* = − 0.11); also for human advisors (std. *β* = − 0.07). Acceptance of false negatives (mistakenly unhealthy) was not influenced by the factors that we considered (*F*_FN_(8, 790) = 30.93, *P* < 0.001, *R*_corr_^2^ = 0.24).

With regards to errors in credit scoring, fewer false negatives were accepted (*F*(5, 1657) = 112.72, *P* < 0.001, *R*_corr_^2^ = 0.25) by older respondents (std. *β* = − 0.06) and those reporting higher household income (std. *β* = − 0.07). The latter also accepted fewer false positives (std. *β* = − 0.06), (*F*(5, 1662) = 88.59, *P* < 0.001, *R*_corr_^2^ = 0.21).

The predictors which were domain-, error-, and algorithm-specific indicated that respondents took the error types into account when asked about algorithm error acceptance. In the last step, we examined the differences in estimations and acceptance of errors made by ADM systems and experts more systematically.

### Experts versus algorithms

For the job domain (suitable applicants), health domain (healthy behavior), and crime domain (recidivism) we analyzed in this section whether estimated and accepted errors vary for ADM and human experts in the role of the advisor (Supplementary Table S4).

A 3 × 2 × 2 ANOVA (advisor x type of error x domain) indicated no general difference (*F*(1, 2446) = 0.14, *P* = 0.707) between expert and algorithm error estimations but that these differed depending on the domain (*F*(1, 4892) = 11.79, *P* < 0.001, *η*_p_^2^ = 0.01) and on the type of error within the domain (*F*(2, 4892) = 32.81, *P* < 0.001, *η*_p_^2^ = 0.01). Lower rates of false negatives (minus 4.6 out of 100 suitable applicants) were estimated for job algorithms (*F*(1, 2622) = 78.50, *P* < 0.001, *η*_p_^2^ = 0.03). In recidivism prediction, there was a statistical interaction of type of error and advisor (*F*(1, 2417) = 4.12, *P* = 0.042, *η*_p_^2^ < 0.01), though there were no meaningful differences (Supplementary Table S4). Minimally higher error rates were estimated for recognizing healthy behavior, where false positives and false negatives were estimated to be 1.1 and 2.0 percentage points higher, respectively, for ADM than for humans (*F*(1, 2567) = 8.87, *P* < 0.001, *η*_p_^2^ < 0.01).

Differences in acceptance of errors made by algorithms or experts depended on the domain (*F*(1, 4896) = 3.08, *P* = 0.046, *η*_p_^2^ < 0.01) and on the type of error within the domain (*F*(1, 4896) = 3.45, *P* = 0.032, *η*_p_^2^ = 0.01) (Supplementary Table S4). There was a generally lower acceptance of algorithm errors in suitability prediction (*F*(1, 2631) = 4.92, *P* = 0.027, *η*_p_^2^ < 0.01); false negatives were also considered less acceptable than false positives in the case of algorithms (F(1, 2631) = 12.06, *P* = 0.001, η_p_^2^ = 0.01). For recidivism prediction, in contrast, differences in acceptance are limited. False positives seem to be considered less acceptable when caused by algorithms than by experts. Mistakenly predicting false recidivism was generally less accepted (*F*(1, 2780) = 4.24, *P* = 0.040*, η*_p_^2^ < 0.01). Notably, it was generally considered less acceptable to falsely diagnose unhealthy behavior than to overlook it (*F*(1, 2575) = 37.63, *P* < 0.001*, η*_p_^2^ = 0.01).

An analysis of factors that lead to ADM-specific acceptance of errors confirms that people above the age of 29 (*F*(3, 2717) = 9.01, *P* < 0.001*, η*_p_^2^ = 0.01) accept a higher number of human than algorithm errors (about 1 to 2 more per 100 predictions), while those younger than 29 accept fewer human errors and more algorithm errors (a difference of about 4 in 100 predictions). Similar distinctions could not be confirmed for gender or for monthly household net income.

Additionally, an exploratory analysis of the relationship between estimations and acceptance, which were strictly correlated (between *r* = 0.27 and 0.43), indicated that error acceptance grows with increasing perception of errors, regardless whether algorithms or experts are presented as advisors (Supplementary Fig. S3).

## Discussion

Our nationwide representative study in Germany delivered two major results. First, the majority of respondents underestimated the error rates of algorithms for credit scoring and recidivism prediction. Those who underestimated the errors were more likely higher educated. A potential cause of these overly positive expectations could lie in a higher exposure to marketing stories and unsubstantiated allegations about AI, as it happened with IBM Watson and Google Flu Trends^47^. Exaggerations concerning predictive accuracy of algorithms are widely spread across non-fiction books, scientific and traditional public media. Future media analyses should examine differences in AI storytelling between those media and those used by audiences of lower socioeconomic status. Focus groups on ADM perceptions^48^ could reveal further conditions of unrealistic perceptions concerning artificial intelligence among higher educated people, so that interventions can be designed to adjust them realistically.

Lower expectations about algorithm accuracy were found among respondents with lower education, less employment, and lower income. Overly negative expectations could indicate total absence of any familiarity and knowledge but may also reflect somewhat justified distrust of new technologies that could affect vulnerable citizen’s everyday life in unwelcome ways (e.g. workplace^49^). Less numerate people are less likely to appreciate algorithms^18^. Also, we cannot exclude a domain-independent general expectation about algorithm accuracy that could explain the larger differential for the less accurate algorithm (recidivism prediction) than for the more accurate algorithm (credit scoring). This calls for research on first-hand experience and training with ADM systems that have to be accessible and comprehensible to people of lower education and that could help adjust performance expectations toward the ground truths, e.g. ballpark correctness of estimated errors.

Second, respondents accepted even fewer algorithm errors than they expected to occur, only 5% to 7% errors compared with their estimates of about 20% to 30% errors. That is, major parts of the public in Germany are not willing to accept the current level of errors of the credit scoring and recidivism systems in question. Also, most of the people estimate that more ADM errors happen in algorithmic predictions of job suitability and health behavior than they are willing to accept. This discrepancy between accepted error rate and both the perceived and actual accuracy of algorithms is striking, and has been a neglected issue in previous studies with a general population^9–15^. Similar to error observations^22^ such expectations may hamper confidence in ADM. This would be in line with the concerns of the US public about the acceptability of algorithms in recidivism prediction and resume screening of job applicants: More than 50% find them unacceptable^16^ and perceive those systems to be incapable of doing nuanced work across individuals.

The discrepancy between the actual, estimated, and accepted levels of errors calls for efforts to improve the various algorithms, with regulatory oversight of ADM quality and fairness. It also calls for more transparency about the actual performance; specifically, decision-makers and those affected by ADM systems need to be informed in an understandable way about ADM performance to enable informed participation.

Anticipated consequences (e.g. costs) of different types of error appear to influence one’s willingness to accept algorithm errors. People with higher household income accept fewer errors in credit scoring. A higher willingness to accept false positives than false negatives in predicting defaulting is noteworthy, given that 98% of inhabitants in Germany are non-defaulters in the credit scoring system and thus can only end up as a false positive, not as a miss. This puzzling result suggests that people fear harms to the economy more than to themselves, given that the proportion of lenders, landlords, and entrepreneurs among the general population is quite limited. People who experienced phases of unemployment in the past ten years appear to be more open for errors in their favor, namely false positives in predicting suitable job candidates. Older people were less accepting of overlooking suitable candidates. Overlooking recidivizing offenders was less accepted by women and people with higher income. Further investigation is needed to clarify why those plausible associations were limited exclusively to ADM perception. One could hypothesize, for instance, that we could detect the associations because algorithms as advisors had amplified the respondent’s preferences to avoid certain consequences.

Furthermore, ADM systems in our study were estimated to perform roughly at par with experts. Here, people’s expectations appear to be in line with meta-analytic results, according to which algorithms were not found to perform better than experts in prediction tasks^50,51^. Exceptions are indicated for the better algorithmic prediction of average academic grade points and job and training performance ratings^52^. Akin to that, our participants expected that algorithms detect suitable job candidates better than experts. In contrast, doctors were perceived to make slightly fewer errors of both types in evaluating health behavior, a perception that is not found elsewhere in the literature. Perceived harm of errors may contribute to domain-specific algorithm aversion in healthcare^38,39,53^.

Because the respondents were willing to accept fewer errors by ADM than by human experts at least in some domains (e.g. disregarding suitable job applicants), the public may likely expect some ADM systems to be more error-free than humans in order to accept these. Here, the uncertainty of a decision problem may play a role. Algorithms perform better under certainty (e.g. geometry) than with problems of risk^54^ (mostly aleatory “irreducible” uncertainty, e.g. dice games or card games), and people accordingly choose algorithms when making decisions under risk^55^. At the same time, algorithms perform better on problems of risk than on problems of uncertainty such as in forecasting human behavior (high epistemic uncertainty). It needs to be experimentally confirmed whether laypeople accordingly accept more errors by ADM systems with increasing epistemic uncertainty.

Contrary to studies that identify risk-seeking preferences to explain the preference of imperfect algorithms over human advice^56^, our representative study did not find any indications of a relationship between risk preference and the acceptance of algorithm errors across three domains. An additional analysis of the asymptomatic growth in error acceptance (toward about 20%, Supplementary Fig. S3) did not indicate diminishing sensitivity to each expected marginal unit of error. Observing additional errors when there have been greater amounts of algorithm errors before makes a smaller subjective difference than when there have been lower amounts^55^. Similarly, expecting additional errors when there have been greater amounts of errors is associated with a smaller increase in error acceptance than when there have been lower amounts. The willingness to accept errors does not increase proportionately with error expectation, neither for algorithm nor expert judgments.

Finally, the lack of familiarity with domain-specific algorithms^22^ may play a substantial role when expectations are formed. The majority of people are not doctors, scoring experts, legal experts, or personnel managers, so direct experience as ADM users is unlikely. Accuracy estimates of participants with potential interaction (repaying a loan, moving to a new flat) with credit scoring were not noticeable. This points to the question of how the general population, which applies for jobs, flats, and credits, will instead develop a sufficient understanding of ADM systems. Providing information about their accuracy and reliability could reduce algorithm aversion^19^, in particular competitive performance information (algorithm vs human)^57^. Both observed and inferred accuracy of predictive algorithms^58,59^, even if it is low^60^, along with confidence information for each prediction^61^, may correct performance expectations and could improve trust in algorithms (although many users trust them independent of performance^62^ and many are less confident when observing badly performing algorithms^22^). Alternatively, more frugal algorithms^63^ could be used. Opposed to just using interpretable models^64^ and explaining complex models^65^, frugal algorithms assure both transparency and understandability (e.g. explaining a prediction^60^ or an error), which are potential measures to correct performance expectations and to increase trust in ADM systems^66^.

Limitations. Although we used an experimental manipulation (between-subjects) within a household survey, the limited time and attention within a period of eight minutes restricted the possibilities to provide more details for the decision-support problems in question. For instance, “being suitable for a job” in applicant screening, which meant that an applicant fulfils the requirements of a job, could have been misconstrued as meaning that the applicant is among the top candidates. To improve valid understanding of the problems, a large-scale experiment with scenarios or videos would be necessary, ideally with repeated measurements of perceptions of expert and algorithm performance within-subjects.

The developers of COMPAS state that the risk scales were designed to identify risk groups, “not a particular high-risk individual” (p. 31)^67^. This contrasts with their actual use for individual classification^5^, as we applied them for our decision-support problem. However, we introduced the developers’ terminology of “predicting the risk of recidivism” (instead of “predicting recidivism”), which may have hampered item comprehension by inviting participants to conclude that not all offenders will (not) recidivize according to the prediction. In that case, participants would have expected and likely accepted more errors. This would imply that our assessment in fact underestimated error expectations and acceptance regarding the prediction of actual recidivism.

Another limitation to the present study is that the estimates for the actual accuracy of the credit scoring algorithm are self-reported by the SCHUFA. The accuracy estimates for COMPAS, in contrast, stem from independent scientific studies. The SCHUFA being a commercial company, one could argue that their estimates of their own algorithms might be inflated. We have no evidence of this possibility, but if it were the case, that would mean that the public overestimates the accuracy of the algorithm to an even greater extent than what we show in Fig. 2a.

Nor can we exclude the possibility that people in Germany have a more precise sense of the accuracy of other ADM systems, as we could only compare respondents’ estimates to the available accuracies for the domains of credit scoring and recidivism prediction. However, given the lack of further learning possibilities or transparent information available for other ADM systems, we have no reason to assume that the overestimates are unique to recidivism and credit scoring.

Currently, ADM systems are spreading across most life domains, including personalized medicine, consumer scoring, credit scoring, insurers’ customer classification, people analytics, learning analytics, and predictive policing. This cultural change requires digital risk literacy on the part of citizens^68^, including the ability to evaluate the performance of predictive algorithms. Accordingly, schools need to teach students the requisite concepts to understand ADM systems, including input, output, consequences, features, feature weights, fairness, and performance-related properties such as false alarm rate, miss rate, positive predictive value, and cross-validation, along with the skills to locate the relevant information.

Yet knowledge of the actual performance that is delivered by credit scoring and recidivism prediction does not suffice. With regards to both algorithms and experts it is important to keep in mind that people are sensitive to different types of error because these present different costs for them and others. Laypeople consider these potential costs when confronted with algorithmic decision support. Educational materials on decision-support systems should thus inform readers about the performance of algorithms. Facilitating this understanding, however, also requires a culture of transparency and trust, cultivated by commercial firms or imposed by regulators, in which people gain easy access to relevant information about the reliability of algorithms in credit scoring, people scoring, and other critical domains^69^.

## Methods

### Data

The study data were collected as a part of the “Innovation Sample” of the German Socio-Economic Panel Study (SOEP IS)^70^ because it is a high-quality sample of the population in Germany, including immigrants and resident foreigners, randomly selected from a set of randomly selected places in Germany. Within this longitudinal survey, which started in 2011, all household members 16 years of age and up are interviewed in person annually, mitigating usual sampling biases due to high age, low affinity for technologies, or vulnerable status. The panel’s representativeness relies on interviewing split-offs (younger panel members founding their own families). Informed consent was obtained from all respondents. Our sample with 3086 respondents (weighted: 52.1% female, M = 50.4 years of age (SD = 19.0)) is representative of the adult population in Germany^71^ (Supplementary Table S5).

### Design

In accordance with an experimental between-subjects design, after responding to credit scoring items, respondents were randomly assigned to either algorithm or expert predictions (recidivism, job suitability, healthiness of behavior). The study was approved by the Institutional Ethics Board of the Max Planck Institute for Human Development (Berlin, Germany). It was carried out in accordance with the guidelines and regulations of the Max Planck Society for the Advancement of Science in Germany and in accordance with the Declaration of Helsinki.

### Measures

We used four partially known scenarios to introduce ADM systems, of which credit scoring is fully established in Germany, recidivism prediction is established outside Germany, and suitability prediction is currently being established in Germany; the evaluation of healthy behavior was derived from established health bonus programs.

Error rates for each algorithm and each expert were assessed with a normalized frequency format for both types of errors: missing targets and false alarms. The algorithms described in the items (see an overview in Supplementary Table S6 online) targeted “whether an applicant is suitable” for a preliminary selection of job applicants and whether an insured person “lives in a healthy manner” for health insurance premiums. Algorithms in credit scoring and recidivism prediction, by contrast, had deviant targets (insolvent people, recidivizing offenders).

To give item examples: In the US judiciary, offenders are regularly reviewed for early release. The computer program COMPAS assesses whether an offender will recidivize and commit another crime within the next 2 years. Now please imagine a group of 100 offenders who are actually at risk of recidivism: How many of them do you think the computer program mistakenly assesses as not being at risk of recidivism? [estimated false negatives]? What would you personally find acceptable: At most, how many of these 100 offenders could be mistakenly assessed as not being at risk of recidivism by the computer program? [accepted false negatives]? Now please imagine a group of 100 offenders who are not at risk of recidivism: How many of them do you think the computer program mistakenly assesses as being at risk of recidivism? [estimated false positives]? What would you personally find acceptable: At most, how many of these 100 offenders could be mistakenly assessed as being at risk of recidivism by the computer program? [accepted false positives]? There are missing values because participants were not forced to respond.

A validated self-report item about risk attitude^32^ was included (11-point). Standard demographic variables from the SOEP survey that we used (see an overview in Supplementary Table S5 online) were gender, age, education and occupation status, household income (per month), event of unemployment (in the last ten years), health insurance status (statutory or private), and self-rated health status (5-point).

### Process

Our segment of the household survey began with an introductory example about security scanners that illustrated the types of errors in classification (Supplementary Table S6). After receiving a short explanation of credit scoring 2760 to 2765 participants responded to error-specific estimation (always first) and 2773 to 2789 responded to acceptance items (always second). The false positive and false negative item pairs were presented in random order. Afterward, half of the participants were assigned to the algorithm condition (“computer programs”), and the other half to the expert condition (e.g. “personnel manager”). Participants were then presented with the scenarios job suitability (1344 to 1360 respondents), recidivism prediction (1311 to 1351 respondents), and healthy behavior (1306 to 1326 respondents) in random order. Within each scenario, after a brief introduction, the false positive or the false negative item pair (estimation first, acceptance second) was presented randomly. Interviewers read all questions aloud to the participants.

### Analysis

Data were analyzed with regression techniques. All data were weighted on the person level to fine-tune for representativeness.

## Supplementary Information

Supplementary Information.

## Data Availability

Data are available from the German Socio-economic Panel Study (SOEP) only because of third-party restrictions (for requests, please contact soepmail@diw.de). The scientific use file of the SOEP with anonymous microdata is made available free of charge to universities and research institutes for research and teaching purposes. The direct use of SOEP data is subject to the strict provisions of German data protection law. Signing a data distribution contract is therefore a precondition for working with SOEP data. This contract can be requested with a form available here: http://www.diw.de/documents/dokumentenarchiv/17/diw_01.c.88926.de/soep_application_contract.pdf. For further information, the SOEP hotline at either soepmail@diw.de or + 49 30 89789- 292 can be contacted.

## References

[1] Burton JW, Stein M-K, Jensen TB (2020). A systematic review of algorithm aversion in augmented decision making. J. Behav. Decis. Mak..

[2] Russell SJ (2019). Human Compatible: Artificial Intelligence and the Problem of Control.

[3] Smith BC (2019). The Promise of Artificial Intelligence: Reckoning and Judgment.

[4] Angwin J, Larson J, Mattu S, Kirchner L (2016). Machine bias. ProPublica.

[5] Dressel J, Farid H (2018). The accuracy, fairness, and limits of predicting recidivism. Sci. Adv.

[6] Kleinberg J, Lakkaraju H, Leskovec J, Ludwig J, Mullainathan S (2018). Human decisions and machine predictions. Q. J. Econ..

[7] Stevenson, M.T. & Doleac, J.L. Algorithmic Risk Assessment in the Hands of Humans. *Available at SSRN* (2019).

[8] Lohninger, T. & Erd, J. SUBMISSION for the report to the UN General Assembly on digital technology, social protection and human rights. (Vienna, 2019).

[9] Araujo T, Helberger N, Kruikemeier S, De Vreese CH (2020). In AI we trust? Perceptions about automated decision-making by artificial intelligence. AI Soc..

[10] Kieslich, K., Keller, B. & Starke, C. AI-Ethics by Design. Evaluating Public Perception on the Importance of Ethical Design Principles of AI. Preprint http://arxiv.org/abs/2106.00326 (2021).

[11] Albarrán, I., Molina, J. M. & Gijón, C. in *ITS Online Event.*

[12] Grzymek, V. & Puntschuh, M. What Europe Knows and Thinks About Algorithms Results of a Representative Survey. Bertelsmann Stiftung eupinions February 2019. (2019).

[13] Zhang, B. & Dafoe, A. Artificial intelligence: American attitudes and trends. *Available at SSRN 3312874* (2019).

[14] Kozyreva, A., Herzog, S., Lorenz-Spreen, P., Hertwig, R. & Lewandowsky, S. Artificial intelligence in online environments: Representative survey of public attitudes in germany. (2020).

[15] Kozyreva A, Lorenz-Spreen P, Hertwig R, Lewandowsky S, Herzog SM (2021). Public attitudes towards algorithmic personalization and use of personal data online: Evidence from Germany, Great Britain, and the United States. Hum. Soc. Sci. Commun..

[16] Smith A (2018). Public Attitudes Toward Computer Algorithms.

[17] Fast, E. & Horvitz, E. in *Proceedings of the AAAI Conference on Artificial Intelligence.*

[18] Logg JM, Minson JA, Moore DA (2019). Algorithm appreciation: People prefer algorithmic to human judgment. Organ. Behav. Hum. Decis. Process..

[19] Castelo N, Bos MW, Lehmann DR (2019). Task-dependent algorithm aversion. J. Mark. Res..

[20] Lee MK (2018). Understanding perception of algorithmic decisions: Fairness, trust, and emotion in response to algorithmic management. Big Data Soc..

[21] Efendic, E., van de Calseyde, P. & Evans, A. Slow decision speed undermines trust in algorithmic (but not human) predictions. *PrePrint* (2019).

[22] Dietvorst BJ, Simmons JP, Massey C (2015). Algorithm aversion: People erroneously avoid algorithms after seeing them err. J. Exp. Psychol. Gen..

[23] Dietvorst B (2016). People reject (superior) algorithms because they compare them to counter-normative reference points. SSRN.

[24] Twyman M, Harvey N, Harries C (2008). Trust in motives, trust in competence: Separate factors determining the effectiveness of risk communication. Judgm. Decis. Mak..

[25] EC. Special Eurobarometer 460 - Attitudes Towards the Impact of Digitisation and Automation on Daily Life. (Brussels, 2017).

[26] Ipsos. Public views of Machine Learning. (2017).

[27] National Tracking Poll #170401. (2017).

[28] Green DM, Swets JA (1966). Signal detection theory and psychophysics.

[29] Haselton MG, Buss DM (2000). Error management theory: A new perspective on biases in cross-sex mind reading. J. Pers. Soc. Psychol..

[30] Mitchell G, Garrett BL (2019). The impact of proficiency testing information and error aversions on the weight given to fingerprint evidence. Behav. Sci. Law.

[31] Shiloh S (2010). An experimental investigation of the effects of acknowledging false negative and false positive errors on clients' cancer screening intentions: The lesser of two evils?. Appl. Psychol. Health Well Being.

[32] Frey R, Pedroni A, Mata R, Rieskamp J, Hertwig R (2017). Risk preference shares the psychometric structure of major psychological traits. Sci. Adv..

[33] Pierson, E. Demographics and discussion influence views on algorithmic fairness. Preprint http://arxiv.org/abs/1712.09124 (2017).

[34] Mossberger K, Tolbert CJ, Stansbury M (2003). Virtual Inequality: Beyond the Digital Divide.

[35] Harari YN (2016). Homo Deus: A Brief History of Tomorrow.

[36] He, K., Zhang, X., Ren, S. & Sun, J. in *Proceedings of the IEEE international conference on computer vision.* 1026–1034.

[37] Ott, M., Choi, Y., Cardie, C. & Hancock, J.T. in *Proceedings of the 49th annual meeting of the association for computational linguistics: Human language technologies-volume 1.* 309–319 (Association for Computational Linguistics).

[38] Longoni C, Bonezzi A, Morewedge CK (2019). Resistance to medical artificial intelligence. J. Consumer Res..

[39] Promberger M, Baron J (2006). Do patients trust computers?. J. Behav. Decis. Mak..

[40] Eastwood J, Snook B, Luther K (2012). What people want from their professionals: Attitudes toward decision-making strategies. J. Behav. Decis. Mak..

[41] Diab DL, Pui S-Y, Yankelevich M, Highhouse S (2011). Lay perceptions of selection decision aids in US and Non-US samples. Int. J. Sel. Assess..

[42] SCHUFA. Zuverlässiger Score. Sichere Bank. - Der Schufa Score für Banken 3.0., (2019).

[43] Brennan T, Dieterich W, Ehret B (2009). Evaluating the predictive validity of the COMPAS risk and needs assessment system. Crim. Justice Behav..

[44] SCHUFA. Kredit Kompass 2019. (2019).

[45] Gigerenzer G, Hoffrage U (1995). How to improve Bayesian reasoning without instruction: Frequency formats. Psychol. Rev..

[46] Demartini, G. & Mizzaro, S. in *European Conference on Information Retrieval.* 488–491 (Springer).

[47] Gigerenzer, G. in *Critical thinking in psychology* (eds R. J. Sternberg & D. F. Halpern) 197–223 (Cambridge University Press, 2020).

[48] Mirowska, A. & Mesnet, L. Preferring the devil you know: Potential applicant reactions to artificial intelligence evaluation of interviews. *Hum. Resour. Manag. J.*10.1111/1748-8583.12393.

[49] Frey CB, Osborne MA (2017). The future of employment: How susceptible are jobs to computerisation?. Technol. Forecast. Soc. Chang..

[50] Ægisdóttir S (2006). The meta-analysis of clinical judgment project: Fifty-six years of accumulated research on clinical versus statistical prediction. Couns. Psychol..

[51] Kaufmann E, Wittmann WW (2016). The success of linear bootstrapping models: Decision domain-, expertise-, and criterion-specific meta-analysis. PLoS ONE.

[52] Kuncel NR, Klieger DM, Connelly BS, Ones DS (2013). Mechanical versus clinical data combination in selection and admissions decisions: A meta-analysis. J. Appl. Psychol..

[53] Shaffer VA, Probst CA, Merkle EC, Arkes HR, Medow MA (2013). Why do patients derogate physicians who use a computer-based diagnostic support system?. Med. Decis. Making.

[54] Knight, F.H. *Risk, uncertainty and profit*. (1964).

[55] Dietvorst BJ, Bharti S (2020). People reject algorithms in uncertain decision domains because they have diminishing sensitivity to forecasting error. Psychol. Sci..

[56] Jay Dietvorst, B. & Bharti, S. in *ACR North American Advances* Vol. 47 (eds Bagchi, R., Block, L., Lee, L. & Duluth) 78–81 (Association for Consumer Research, 2019).

[57] Jussupow, E., Benbasat, I. & Heinzl, A. Why are we averse towards Algorithms? A comprehensive literature Review on Algorithm aversion. (2020).

[58] Yin, M., Wortman Vaughan, J. & Wallach, H. in *Proceedings of the 2019 CHI Conference on Human Factors in Computing Systems.* 1–12.

[59] Yu, K., Berkovsky, S., Taib, R., Zhou, J. & Chen, F. in *Proceedings of the 24th International Conference on Intelligent User Interfaces.* 460–468.

[60] Lai, V. & Tan, C. in *Proceedings of the Conference on Fairness, Accountability, and Transparency.* 29–38.

[61] Zhang, Y., Liao, Q.V. & Bellamy, R. K. Effect of Confidence and Explanation on Accuracy and Trust Calibration in AI-Assisted Decision Making. Preprint http://arxiv.org/abs/2001.02114 (2020).

[62] Springer, A., Hollis, V. & Whittaker, S. in *2017 AAAI Spring Symposium Series.*

[63] Hafenbrädl S, Waeger D, Marewski JN, Gigerenzer G (2016). Applied decision making with fast-and-frugal heuristics. J. Appl. Res. Mem. Cogn..

[64] Poursabzi-Sangdeh, F., Goldstein, D.G., Hofman, J.M., Vaughan, J.W. & Wallach, H. Manipulating and measuring model interpretability. Preprint http://arxiv.org/abs/1802.07810 (2018).

[65] Cheng, H.-F. *et al.* in *Proceedings of the 2019 CHI Conference on Human Factors in Computing Systems.* 1–12. 10.1145/3290605.3300777 PMC6800573 31633126

[66] Ribeiro, M.T., Singh, S. & Guestrin, C. in *Proceedings of the 22nd ACM SIGKDD international conference on knowledge discovery and data mining.* 1135–1144.

[67] Northpointe. Practitioner’s Guide to COMPAS Core. (Northpointe, 2015).

[68] Gigerenzer, G. *Risk savvy: How to make good decisions*. (Penguin, 2015).

[69] O’Neill O (2018). Linking trust to trustworthiness. Int. J. Philos. Stud..

[70] Richter D, Schupp J (2015). The SOEP Innovation Sample (SOEP IS). Schmollers Jahrbuch: Journal of Applied Social Science Studies/Zeitschrift für Wirtschafts-und Sozialwissenschaften.

[71] Goebel J (2019). The German socio-economic panel (SOEP). Jahrbücher für Nationalökonomie und Statistik.

