# Supplementary material for: People underestimate the errors made by algorithms for credit scoring and recidivism prediction but accept even fewer errors
Source: Sci Rep. 2021 Oct 11;11:20171. doi: 10.1038/s41598-021-99802-y (PMC8505498; doi:10.1038/s41598-021-99802-y)
Supplement: Supplementary file 1 — Supplementary Information. [file 41598_2021_99802_MOESM1_ESM.pdf]

Supplementary Information for

People underestimate the errors made by algorithms for credit scoring and recidivism prediction but accept even fewer errors

Felix G. Rebitschek[a,b,1]
Gerd Gigerenzer [a, b]
Gert G. Wagner[a,b,c]

[a]Harding Center for Risk Literacy, Faculty of Health Sciences Brandenburg, University of Potsdam, Potsdam
[b]Max Planck Institute for Human Development, Berlin
[c]German Socio-Economic Panel Study (SOEP), Berlin

[1]Felix G. Rebitschek, **Email:** rebitschek@mpib-berlin.mpg.de

This PDF file includes:

Figures S1a-d to S3
Tables S1 to S8

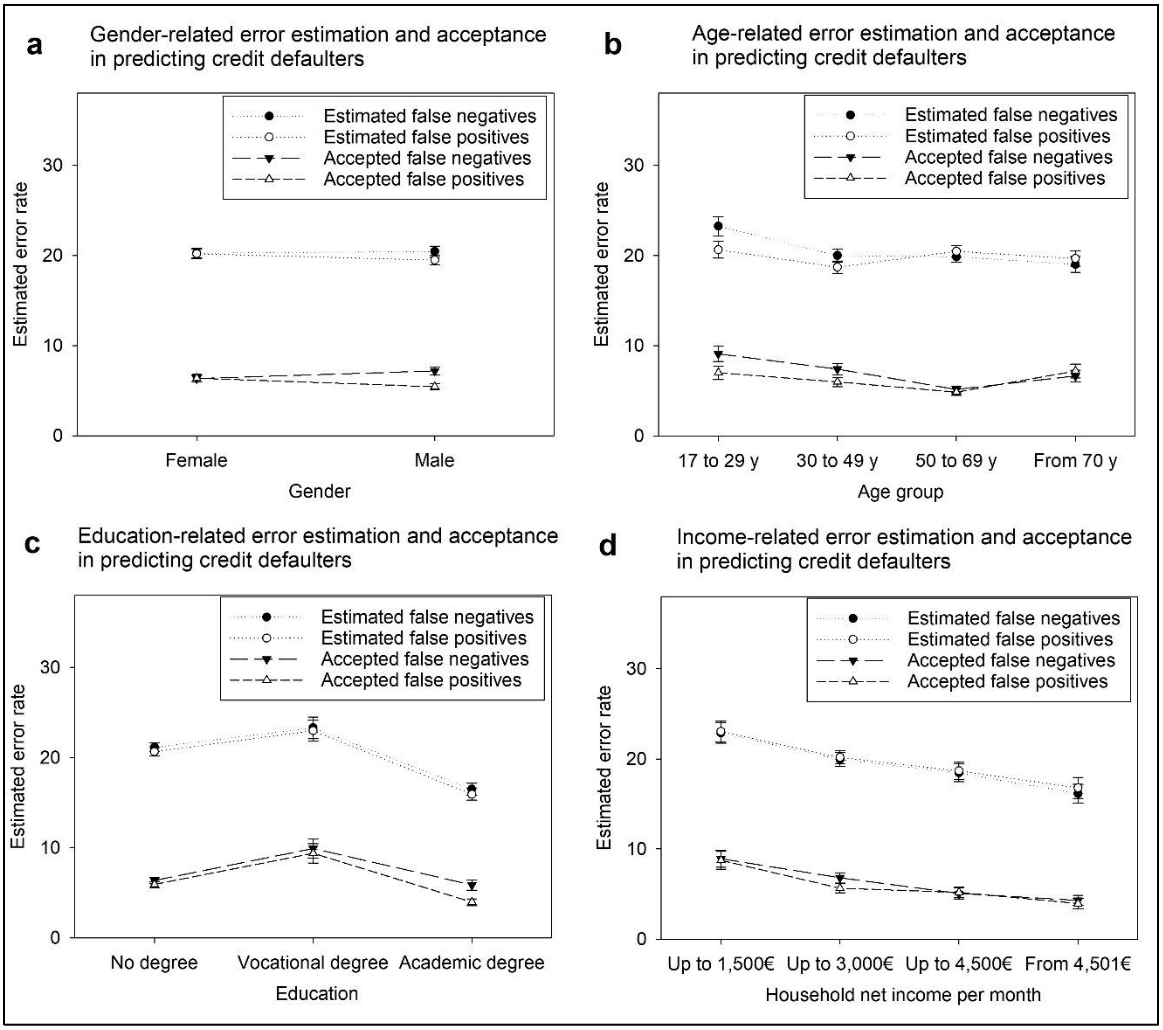

Figures S1a-d: Relationships between sample characteristics and error estimations and acceptance in predicting credit defaulting. Error bars show the standard error of the mean.

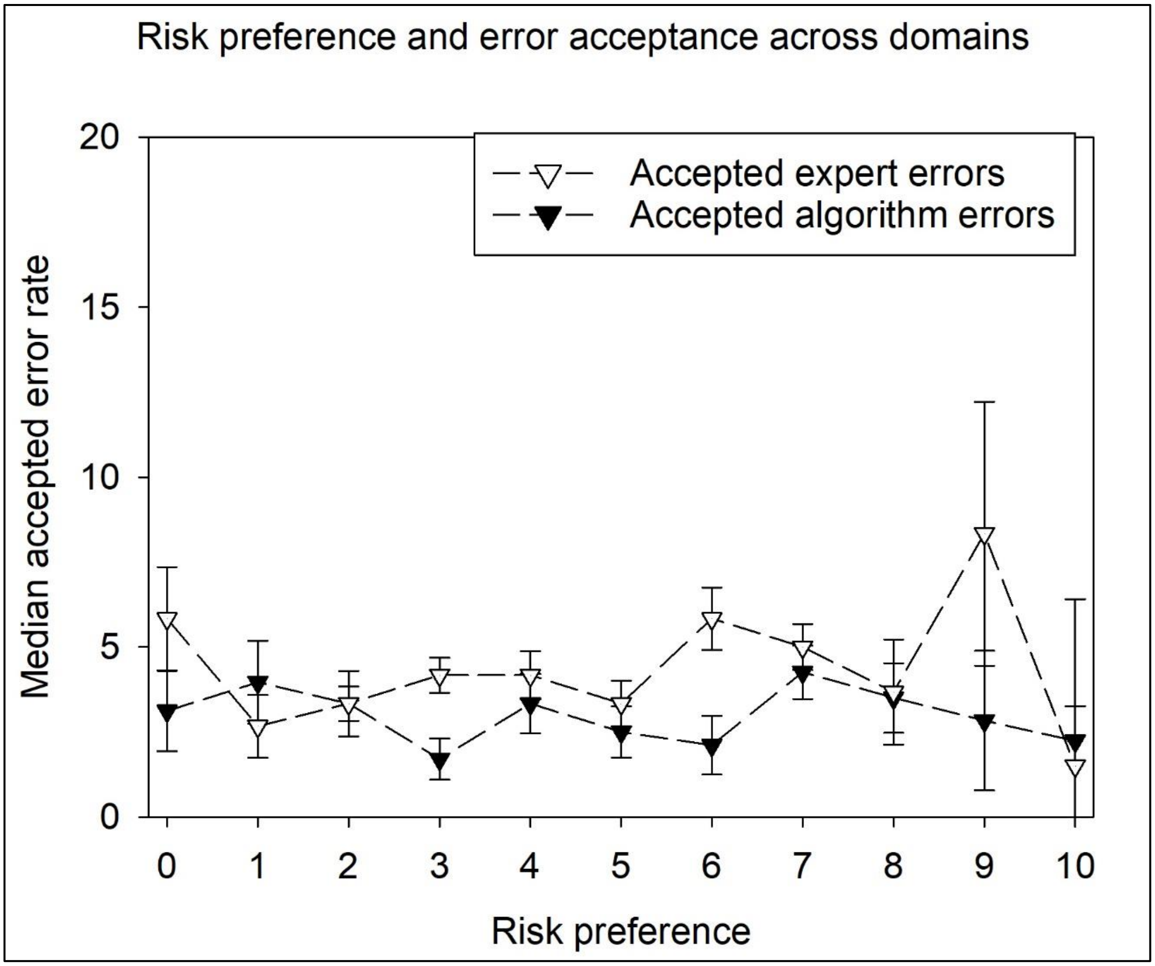

Figure S2. Median acceptance of algorithm and expert errors depending on risk preference. Sample is weighted. Error bars show the standard error of the mean.

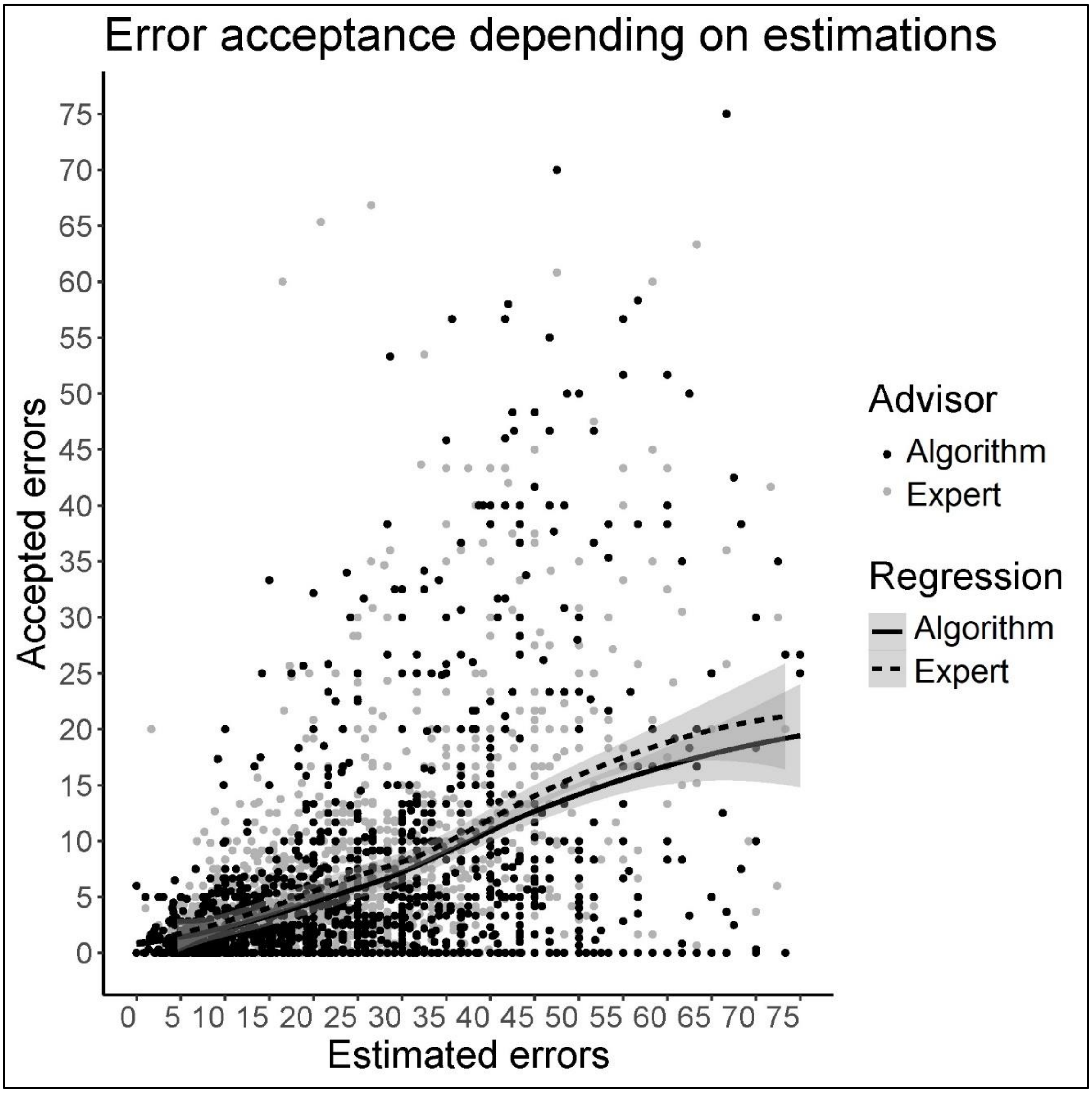

Figure S3: The scatterplot shows each respondent's estimates and acceptance of errors. Each point corresponds to one respondent (gray for expert condition, black for the algorithm condition). The lines show local regressions. The gray shadings show the standard error of the mean.

Table S1. Percentage of respondents who assessed the corresponding system to be error-free in terms of predictions (illusion of certainty).

		**Algorithms**		**Experts**
	N	Error-free [%]	N	Error-free [%]
Mistakenly suitable applicant (false positive)	1,380	2.9%	1,318	3.6%
Mistakenly unsuitable applicant (false negative)	1,374	3.1%	1,324	1.5%
Mistakenly not at risk of recidivism (false negative)	1,361	2.6%	1,291	1.6%
Mistakenly at risk of recidivism (false positive)	1,348	2.1%	1,275	1.7%
Mistakenly behaving healthily (false positive)	1,333	4.7%	1,298	3.2%
Mistakenly behaving unhealthily (false negative)	1,343	4.6%	1,299	4.3%
Mistakenly not creditworthy (false positive)	2,733	4.0%		
Mistakenly creditworthy (false negative)	2,728	5.9%		

Table S2. Distribution of characteristics according to estimation (respondents grouped by five percent of scale value around the ground truth) and acceptance of credit scoring errors (presented as accuracy; grouped around AUC = .90, distinguishing highly critical respondents and less critical respondents along a lay-comprehensible border [1 error per 10 assignments]).

Credit defaulting prediction<br>Characteristic		Underest. accuracy AUC<.76 n=850	Correctly est. accuracy AUC=.81±.05 n=552	Overest. accuracy AUC>.86 n=1,172	*p* [1]	Accepting AUC<.90 n=364	Accepting AUC≥.90 n=2,229	*p* [1]
Gender [%]	Female	52.6	52.3	51.7	.920	53.1	52.2	.821
	Male	47.4	47.7	48.3		46.9	47.8	
Age [M (SD)] in years		49.2 (18.2)	50.5 (18.5)	50.1 (17.9)	.403	47.9 (19.1)	50.2 (17.9)	.025
Household net income [M (SD)] in EUR		2,653 (1,922)	3,171 (1,814)	3,090 (1,890)	<.001	2,462 (1,647)	3,044 (1,919)	<.001
Education Diplomas/degrees from secondary/tertiary school [%]	Sec. Gen. School Leaving Cert.	30.0	20.2	17.7	<.001	30.9	20.5	<.001
	Intermediate School Degree	31.1	31.5	29.7		32.6	30.4	
	Leaving Cert. From Voc. HS	6.6	6.5	8.5		4.6	7.9	
	College Entrance Exam	19.6	30.0	31.7		15.3	29.3	
	Other	7.7	9.6	10.3		7.6	9.8	
	Dropout, No School Certificate	3.6	0.9	1.3		6.5	1.3	
	Currently In School	1.3	1.4	.8		2.5	0.9	
Vocational degree attained [%]	Apprenticeship	40.4	43.4	40.3	.014	37.6	41.7	.084
	Vocational School	9.0	9.9	9.4		8.6	9.4	
	Health Care School	0.0	0.1	0.1		0.1	0.1	
	Technical School	7.5	5.4	4.1		5.8	5.5	
	Civil Servant Training	1.5	3.0	2.4		1.0	2.3	
	Other Degree	1.3	0.2	1.9		1.7	1.4	
	Completed Voc. Training/Edu.	3.5	2.4	3.2		5.4	2.8	
	NA (e.g. college degree)	36.8	35.6	38.6		39.8	36.9	
Completed college education [%]	FH (Univ. of Applied Sciences)	4.3	6.9	7.9	<.001	5.9	7.2	.002
	University, Technical College	7.9	10.5	13.9		6.6	11.9	
	College Not In Germany	0.1	0.0	0.2		0.0	0.1	
	Engineering, Technical School	0.9	1.8	1.4		0.2	1.6	
	University (East)	2.0	0.7	1.8		0.7	1.8	
	Graduation, state doctorate	2.7	2.2	3.7		1.7	2.9	
	Not applicable	82.1	77.8	71.0		84.9	74.5	

Occupation status [%]	Full time employment	35.3	40.4	40.5	<.001	28.2	40.4	<.001
	Part-time employment	11.3	14.4	15.4		11.8	14.1	
	Training/apprenticeship	3.1	2.7	1.7		4.0	2.0	
	Marginally employed	4.8	6.7	5.8		6.0	5.6	
	Partial retirement	0.0	0.4	0.1		0.0	0.0	
	Voluntary service	0.2	0.0	0.0		0.5	0.0	
	Workshop for the disabled	0.4	0.0	0.1		0.0	0.1	
	Not employed (anymore)	45.0	35.4	36.3		49.5	37.6	
Unemployment phases in the past ten years [%][2]	Yes	31.5	22.0	24.0	.001	35.1	25.0	.001
	No	68.5	78.0	76.0		64.9	75.0	
Body Mass Index (BMI) [M (SD)]		27.1 (10.4)	26.4 (7.5)	25.9 (4.8)	.005	27.7 (16.4)	26.2 (4.9)	.001
Self-reported health state [%][2]	Very good	10,4	14,0	12,8	<.001	10,9	12,5	.001
	Good	35,9	40,2	45,1		32,7	42,4	
	Satisfactory	35,8	30,8	29,4		40,8	30,3	
	Not so good	14,1	12,8	10,5		13,1	12,0	
	Poor	3,8	2,1	2,2		2,5	2,9	
Health insurance status [%][2]	Statutory health insurance	90.5	88.5	84.7	<.001	94.7	86.5	<.001
	Private health insurance	9.5	11.5	15.3		5.3	13.5	
Risk preference [M (SD)] from 0 (avoidance) to 10 (preference)		4.6 (2.5)	4.2 (2.2.)	4.5 (2.4)	.014	4.5 (2.6)	4.4 (2.3)	.540

1 Variance analyses and Chi^2-tests, respectively
2 Subsamples were presented with the items

Table S3. Distribution of characteristics according to estimation (respondents grouped by five percent of scale value around the ground truth) and acceptance of algorithm errors in recidivism prediction (presented as accuracy; grouped around AUC = .90, distinguishing highly critical respondents and less critical respondents along a lay-comprehensible border [1 error per 10 assignments]).

Recidivism prediction<br>Characteristic		Underest. accuracy AUC<.63 n=314	Correctly est. accuracy AUC=.68±.05 n=178	Overest. accuracy AUC>.73 n=770	*p* [1]	Accepting AUC<.90 n=155	Accepting AUC≥.90 n=1,130	*p* [1]
Gender [%]	Female	49.3	52.6	52.0	.704	44.7	52.2	.086
	Male	50.7	47.4	48.0		55.3	47.8	
Age [M (SD)] in years		49.5 (18.3)	53.2 (18.6)	47.7 (18.0)	.001	48.5 (19.6)	49.1 (18.2)	.713
Household net income [M (SD)] in EUR		2,667 (1,793)	2,992 (1,583)	3,136 (1,850)	.012	2,338 (1,390)	3,098 (1,846)	<.001
Education Diplomas/degrees from secondary/tertiary school [%]	Sec. Gen. School Leaving Cert.	25.0	28.6	17.1	<.001	27.8	20.0	<.001
	Intermediate School Degree	38.9	23.3	27.0		22.1	30.2	
	Leaving Cert. From Voc. HS	6.0	8.3	8.2		8.6	7.5	
	College Entrance Exam	16.8	26.3	33.2		17.5	29.8	
	Other	9.7	13.0	10.4		13.8	9.9	
	Dropout, No School Certificate	2.2	0.2	2.2		7.7	1.2	
	Currently In School	1.4	0.4	1.9		2.7	1.4	
Vocational degree attained [%]	Apprenticeship	44.0	40.1	37.1	.012	30.7	40.3	.005
	Vocational School	8.9	10.9	7.9		7.1	8.7	
	Health Care School	0.0	0.0	0.0		0.0	0.0	
	Technical School	4.6	4.5	4.6		6.0	4.9	
	Civil Servant Training	1.5	2.2	2.5		1.9	2.2	
	Other Degree	0.1	3.2	1.8		0.9	1.6	
	Completed Voc. Training/Edu.	7.0	2.8	3.3		9.7	3.4	
	NA (e.g. college degree)	33.9	36.3	42.9		43.7	38.9	
Completed college education [%]	FH (Univ. of Applied Sciences)	4.8	2.2	9.1	<.001	9.5	6.6	.102
	University, Technical College	10.0	6.5	13.1		13.9	11.4	
	College Not In Germany	0.1	0.9	0.0		0.0	0.2	
	Engineering, Technical School	0.8	1.6	1.7		0.0	1.6	
	University (East)	0.4	2.2	2.2		0.8	1.9	
	Graduation, state doctorate	0.9	5.8	3.9		0.5	3.8	
	Not applicable	83.1	80.9	70.0		75.3	74.5	

Occupation status [%]	Full time employment	38.7	29.2	44.1	<.001	34.0	41.1	<.001
	Part-time employment	9.3	18.8	12.0		9.3	13.2	
	Training/apprenticeship	4.4	1.8	2.3		5.9	2.4	
	Marginally employed	4.4	5.7	7.7		6.0	6.3	
	Partial retirement	0.2	0.0	0.2		1.6	0.0	
	Voluntary service	0.0	0.0	0.0		0.0	0.0	
	Workshop for the disabled	0.4	0.2	0.0		0.8	0.0	
	Not employed (anymore)	42.7	44.3	33.6		42.5	36.9	
Unemployment phases in past 10 years [%][2]	Yes	34.4	23.3	28.3	.098	40.4	26.3	.003
	No	65.6	76.7	71.7		59.6	73.7	
Body Mass Index (BMI) [M (SD)]		26.4 (5.0)	27.1 (5.2)	26.1 (6.9)	.137	26.6 (8.2)	25.9 (5.3)	.401
Self-reported health state [%][2]	Very good	8.6	9.0	15.4	<.001	10.8	13.2	.003
	Good	35.0	37.1	42.0		30.5	40.6	
	Satisfactory	40.2	34.5	30.9		47.4	31.3	
	Not so good	12.7	15.8	9.9		9.9	12.3	
	Poor	3.5	3.6	1.8		1.4	2.6	
Health insurance status [%][2]	Statutory health insurance	89.3	87.3	87.7	.669	90.9	87.8	.290
	Private health insurance	10.7	12.7	12.3		9.1	12.2	
Risk preference [M (SD)] from 0 (avoidance) to 10 (preference)		4.8 (2.5)	4.4 (2.2)	4.4 (2.4)	.089	4.1 (2.6)	4.5 (2.4)	.050

1 Variance analyses and Chi^2-tests, respectively
2 Subsamples were presented with the items

Table S4. Overview of domain-specific comparisons of respondents' error estimations and acceptance of algorithms and experts (u-Tests, adjusted alpha/(k-1 = .01); effect size is Cohen's *d*).

	Estimated errors of algorithms over experts		Accepted errors of algorithms over experts	
	d	*p*	*d*	*p*
Mistakenly suitable applicant (false positive)	-	.013	-0.01	<.001
Mistakenly unsuitable applicant (false negative)	-0.11	<.001	-0.08	<.001
Mistakenly predicted recidivism (false positive)	-	.766	-0.01	<.001
Mistakenly predicted no recidivism (false negative)	-	.107	-	.017
Mistakenly behaving healthily (false positive)	-	.119	-	.458
Mistakenly behaving unhealthily (false negative)	0.09	<.001	-	.019

Table S5. Characteristics of the household sample (unweighted data) and split according to the experimental manipulation (algorithm, expert).

Characteristic		All	Algorithm condition	Expert condition	Adult population Germany 2019[1]
		N=3,086	n=1,574	n=1,512	83 Million
Gender [%]	Female	53.8	53.9	53.6	51.1
	Male	46.2	46.1	46.4	48.9
Age [M (SD), Median] in years		54.7 (18.6), 56	54.6 (19.0), 57	54.9 (18.2), 56	51.1
Household net income [M (SD), Median] in EUR		2,822 (1,806), 2,400	2,877 (1,848), 2,500	2,766 (1,761), 2,400	2175, 1960[2]
Education Diplomas/degrees from secondary/tertiary school [%]	Sec. Gen. School Leaving Certificate	28.1	27.8	28.5	28.6
	Intermediate School Degree	31.1	30.8	31.3	23.5
	Leaving Certificate From Voc High School	6.6	6.5	6.8	-
	College Entrance Exam	24.2	25.0	23.4	33.5
	Other	7.6	7.4	7.8	6.7
	Dropout, No School Certificate	1.5	1.5	1.5	4.0
	Currently In School	0.8	1.0	0.7	3.5
Vocational degree attained [%]	Apprenticeship	45.1	44.9	45.3	47.1
	Vocational School	9.7	9.2	10.3	9.3
	Health Care School	0.2	0.1	0.3	-
	Technical School	6.0	5.6	6.4	-
	Civil Servant Training	2.7	2.9	2.6	-
	Other Degree	1.2	1.3	1.1	-
	Completed Vocational Training/Education	2.1	2.5	1.8	-
	Not applicable (e.g. college degree)	33.0	33.6	32.3	43.6
Completed college education [%]	Fachhochschule (Univ. of Applied Sciences)	6.2	5.8	6.6	-
	University, Technical College	10.0	10.2	9.8	17.3
	College Not In Germany	0.3	0.5	0.2	-
	Engineering, Technical School	1.4	1.6	1.2	-
	University (East)	2.1	2.5	1.8	-
	Graduation, state doctorate	2.1	2.2	2.1	1.2
	Not applicable	77.8	77.3	78.3	81.5

Occupation status [%]	Full time employment	32.5	32.6	32.5	32.4
	Part-time employment	12.2	11.2	13.2	13.1
	Training/apprenticeship	1.7	1.8	1.6	1.6
	Marginally employed	5.5	5.7	5.3	5.8[3]
	Partial retirement	0.2	0.2	0.1	0.3[4]
	Voluntary service	0.2	0.1	0.2	0.1[5]
	Workshop for the disabled	0.3	0.3	0.3	0.3[6]
	Not employed (anymore)	47.5	48.1	46.9	46.5
Unemployment phases in past 10 years [%]*	Yes	24.8	25.0	24.7	20.9[7]
	No	75.2	75.0	75.3	79.1
Body Mass Index (BMI) [M (SD), Median]		26.6 (6.6), 25.7	26.6 (6.8), 25.7	26.6 (6.5), 25.8	-
Self-reported health state [%]*	Very good	9.5	9.9	9.1	-
	Good	38.9	38.1	39.8	
	Satisfactory	33.4	33.9	32.9	
	Not so good	14.4	14.8	14.0	
	Poor	3.7	3.3	4.2	
Health insurance status [%]*	Statutory health insurance	87.0	87.0	87.0	89.3
	Private health insurance	13.0	13.0	13.0	10.7
Risk preference [M (SD), Median] from 0 (avoidance) to 10 (preference)		4.3 (2.3), 4	4.3 (2.4), 4	4.2 (2.3), 4	-

* Subsamples were presented with the items
[1] Data source (if not specified else): Federal Statistal Office Germany, Destatis.de
[2] Data source: Eurostat-Database, https://ec.europa.eu/eurostat/de/
[3] Data source: Federal Employment Agency
[4] Data source: German Pension Insurance
[5] Data source: Federal Office for Family and Civil Society Tasks
[6] Data source: Employer's Liability Insurance Association for Health Services and Welfare Care, Data from 2017
[7] Data from 2014

Table S6. Items

<table>
<tr><th>Section</th><th>Algorithm condition group</th><th>Expert condition group</th></tr>
<tr><td>Introduction</td><td colspan="2">In the following, we would like to talk about the errors that can happen in the assessment of human behavior. It is important to know: every assessment, every test, can always make two kinds of errors!
Take as an example the detectors or scanners in the security area at the airport, which check whether someone is carrying a weapon.
Go through them, and then the detector beeps even though you are not carrying anything - then the detector has made an error - it has given a false alarm.
The other errors the detector can make would be if a terrorist passes through and there is no beeping despite the weapon. Then the detector would have missed the weapon.
These two types of errors - false alarm and miss - occur in every assessment, every test.</td></tr>
<tr><td>Credit scoring</td><td colspan="2">You have probably heard of the Schufa. The Schufa estimates the solvency of all persons in Germany. This helps entrepreneurs and landlords to decide whether someone gets a mobile phone contract, an apartment or a loan, for example.

Now please imagine a group of 100 people who are actually insolvent:
How many of them do you think are mistakenly assessed by the Schufa as being solvent?
_____person(s)

What would be acceptable to you: At most, how many of these 100 people could be mistakenly assessed by the Schufa as being solvent?
_____person(s)

Now please imagine a group of 100 people who are actually solvent:
How many of them do you think are mistakenly assessed by the Schufa as being insolvent?
_____person(s)

What would you find acceptable: At most, how many of these 100 people could be mistakenly assessed by the Schufa as insolvent?
_____person(s)</td></tr>
</table>

Suitability prediction	Employers in Germany obtain information about applicants from various sources in order to make a preliminary selection. There are computer programs that use certain criteria such as grades or work experience to assess whether an applicant is suitable and will be considered.<br><br>Now please imagine a group of 100 applicants who are actually unsuitable:<br>How many of them do you think are mistakenly assessed as suitable by computer programs?<br>_____ Applicants<br><br>What would you personally find acceptable: At most, how many of these 100 applicants could be mistakenly assessed as suitable by computer programs?<br>_____ Applicants<br><br>Now please imagine a group of 100 applicants who are actually suitable:<br>How many of them do you think are mistakenly assessed as unsuitable by computer programs?<br>_____Applicants<br><br>What would you personally find acceptable: At most, how many of these 100 applicants could be mistakenly assessed as unsuitable by computer programs?<br>_____ Applicants	Employers in Germany obtain information about applicants from various sources in order to make a preliminary selection. An expert, e.g. a personnel manager, uses certain criteria such as grades or work experience to assess whether an applicant is suitable and will be considered.<br><br>Now please imagine a group of 100 applicants who are actually unsuitable:<br>How many of them do you think are mistakenly assessed as suitable by a personnel manager?<br>_____ Applicants<br><br>What would you personally find acceptable: At most, how many of these 100 applicants could be mistakenly assessed as suitable by a personnel manager?<br>_____ Applicants<br><br>Now please imagine a group of 100 applicants who are actually suitable:<br>How many of them do you think are mistakenly assessed as unsuitable by a personnel manager?<br>_____Applicants<br><br>What would you personally find acceptable: At most, how many of these 100 applicants could be mistakenly assessed as unsuitable by a personnel manager?<br>_____ Applicants

Recidivism prediction	In the US judiciary, offenders are regularly reviewed for early release. The computer program COMPAS assesses whether an offender will recidivize and commit another crime within the next 2 years.<br><br>Now please imagine a group of 100 offenders who are actually at risk of recidivism:<br>How many of them do you think the computer program mistakenly assesses as not being at risk of recidivism?<br>_____offenders<br><br>What would you personally find acceptable: At most, how many of these 100 offenders could be mistakenly assessed as not being at risk of recidivism due to the computer program?<br>_____offenders<br><br>Now please imagine a group of 100 offenders who are actually not at risk of recidivism:<br>How many of them do you think the computer program mistakenly assesses as being at risk of recidivism?<br>_____offenders<br><br>What would you personally find acceptable: At most, how many of these 100 offenders could be mistakenly assessed as being at risk of recidivism due to the computer program?<br>_____offenders	In the US judiciary, offenders are regularly reviewed for early release. An expert, e.g. a legal expert, assesses whether an offender will recidivize and commit another crime within the next 2 years.<br><br>Now please imagine a group of 100 offenders who are actually at risk of recidivism:<br>How many of them do you think the legal expert mistakenly assesses as not being at risk of recidivism?<br>_____offenders<br><br>What would you personally find acceptable: At most, how many of these 100 offenders could be mistakenly assessed as not being at risk of recidivism due to the legal expert?<br>_____offenders<br><br>Now please imagine a group of 100 offenders who are actually not at risk of recidivism:<br>How many of them do you think the legal expert mistakenly assesses to be at risk of recidivism?<br>_____offenders<br><br>What would you personally find acceptable: At most, how many of these 100 offenders could be mistakenly assessed as being at risk of recidivism due to the legal expert?<br>_____offenders

Health behavior	Private health insurers in Germany can take into account the personal responsibility of their insured customers to decide how much they have to pay. A computer program can assess whether an insured person is behaving in a healthy manner.<br><br>Now please imagine a group of 100 insured people who actually behave in an unhealthy way:<br>How many of them do you think are mistakenly assessed by a computer program as behaving in a healthy manner?<br>_____ Insured<br><br>What would you personally find acceptable: a maximum of how many of these 100 insured persons could be mistakenly assessed by a computer program as behaving in a healthy manner?<br>_____ Insured<br><br>Now please imagine a group of 100 insured people who actually behave in a healthy manner:<br>How many of them do you think are mistakenly assessed by a computer program as behaving in an unhealthy manner?<br>____ Insured<br><br>What would you personally find acceptable: a maximum of how many of these 100 insured persons could be mistakenly assessed by a computer program as behaving in an unhealthy manner?<br>_____ Insured	Private health insurers in Germany can take into account the personal responsibility of their insured customers to decide how much they have to pay. An expert, e.g. a doctor, can assess whether an insured person is behaving in a healthy manner.<br><br>Now please imagine a group of 100 insured people who actually behave in an unhealthy way:<br>How many of them do you think are mistakenly assessed by a doctor as behaving in a healthy manner?<br>_____ Insured<br><br>What would you personally find acceptable: a maximum of how many of these 100 insured persons could be mistakenly assessed by a doctor as behaving in a healthy manner?<br>_____ Insured<br><br>Now please imagine a group of 100 insured people who are actually behave in a healthy manner:<br>How many of them do you think are mistakenly assessed by a doctor as behaving in an unhealthy manner?<br>_____ Insured<br><br>What would you personally find acceptable: a maximum of how many of these 100 insured persons could be mistakenly assessed by a doctor as behaving in an unhealthy manner?<br>_____ Insured

Table S7. Distribution of characteristics according to estimation (respondents grouped by quantiles) and acceptance of algorithm errors in predicting suitability of an applicant (presented as accuracy; grouped around AUC = .90, distinguishing highly critical respondents and less critical respondents along a lay-comprehensible border [1 error per 10 assignments]).

Suitability prediction of applicants<br>Characteristic		Quantile$_{33}$: Est. AUC <= .700<br>n=472	Quantile$_{67}$ - Quantile$_{33}$: Est. AUC <= .875<br>n=420	Quantile$_{100}$ - Quantile$_{67}$: Est. AUC > .875<br>n=404	*p* [1]	Accepting AUC<.90<br>n=212	Accepting AUC≥.90<br>n=1,097	*p* [1]
Gender [%]	Female	52.0	53.2	50.2	.692	50.2	53.1	.453
	Male	48.0	46.8	49.8		49.8	46.9	
Age [M (SD)] in years		49.1 (17.9)	49.3 (17.9)	48.0 (18.7)	.549	47.8 (18.9)	49.3 (18.0)	.260
Household net income [M (SD)] in EUR		2,835 (1,641)	3,176 (1,900)	3,038 (1,882)	.086	2,501 (1,330)	3,105 (1,872)	<.001
Education Diplomas/degrees from secondary/tertiary school [%]	Sec. Gen. School Leaving Certificate	25.3	16.2	20.0	.025	23.3	20.2	<.001
	Intermediate School Degree	32.3	28.5	25.8		31.7	28.5	
	Leaving Certificate From Voc HS	5.9	9.1	7.6		6.6	7.6	
	College Entrance Exam	24.9	29.4	31.8		17.2	30.4	
	Other	8.9	12.4	10.9		13.0	10.6	
	Dropout, No School Certificate	1.7	2.5	2.1		6.2	1.3	
	Currently In School	1.1	1.9	1.8		2.0	1.4	
Vocational degree attained [%]	Apprenticeship	40.6	34.4	40.5	.022	34.0	39.5	.131
	Vocational School	9.7	8.1	8.2		10.3	8.9	
	Health Care School	0.1	0.0	0.0		0.1	0.0	
	Technical School	4.1	8.3	3.4		0.0	4.9	
	Civil Servant Training	1.6	3.5	1.1		1.2	2.2	
	Other Degree	0.6	2.2	1.9		2.5	1.3	
	Completed Voc. Training/Education	4.3	3.1	4.9		7.7	3.4	
	Not applicable (e.g. college degree)	39.0	40.3	40.1		38.9	39.8	
Completed college education [%]	FH (Univ. of Applied Sciences)	5.3	10.0	4.7	.016	7.1	6.6	.479
	University, Technical College	11.4	12.0	12.4		11.0	12.2	
	College Not In Germany	0.0	0.1	0.5		0.0	0.2	
	Engineering, Technical School	1.3	2.0	1.1		0.4	1.6	
	University (East)	1.4	1.8	1.4		0.8	1.7	
	Graduation, state doctorate	3.4	4.9	1.7		2.0	3.7	
	Not applicable	77.2	69.3	78.1		78.8	73.9	

Occupation status [%]	Full time employment	39.0	41.8	41.7	.430	35.4	41.0	.029
	Part-time employment	14.3	11.5	12.1		11.3	13.7	
	Training/apprenticeship	3.8	2.4	2.2		6.1	2.2	
	Marginally employed	4.6	7.8	6.1		5.6	6.4	
	Partial retirement	0.1	0.0	0.5		0.0	0.2	
	Voluntary service	0.0	0.0	0.0		0.0	0.0	
	Workshop for the disabled	0.2	0.0	0.1		0.0	0.1	
	Not employed (anymore)	37.9	36.6	37.3		41.6	36.3	
Unemployment phases in past 10 years [%][2]	Yes	32.3	29.5	23.1	.044	39.0	26.5	.004
	No	67.7	70.5	76.9		61.0	73.5	
Body Mass Index (BMI) [M (SD)]		26.6 (8.2)	26.3 (4.8)	25.7 (4.8)	.110	26.3 (10.7)	26.2 (5.0)	.786
Self-reported health state [%][2]	Very good	8.8	10.2	18.4	.001	10.7	12.8	.002
	Good	39.0	42.4	38.6		33.7	41.2	
	Satisfactory	38.2	31.5	31.2		44.7	31.1	
	Not so good	11.2	13.5	10.1		10.3	12.1	
	Poor	2.8	2.5	1.8		0.6	2.8	
Health insurance status [%][2]	Statutory health insurance	89.1	86.9	88.7	.539	94.9	87.0	.001
	Private health insurance	10.9	13.1	11.3		5.1	13.0	
Risk preference [M (SD)] from 0 (avoidance) to 10 (preference)		4.6 (2.5)	4.4 (2.4)	4.5 (2.4)	.449	4.6 (2.7)	4.4 (2.4)	.486

1 Variance analyses and Chi^2-tests, respectively
2 Subsamples were presented with the items

Table S8. Distribution of characteristics according to estimation (respondents grouped by quantiles) and acceptance of algorithm errors in predicting healthy behavior (presented as accuracy; grouped around AUC = .90, distinguishing highly critical respondents and less critical respondents along a lay-comprehensible border [1 error per 10 assignments]).

Suitability prediction of applicants<br>Characteristic		$Quantile_{33}$: Estimating AUC <= .725<br>n=431	$Quantile_{67}$ - $Quantile_{33}$: Estimating AUC <= .900<br>n=543	$Quantile_{100}$ - $Quantile_{67}$: Estimating AUC > .900<br>n=289	*p* [1]	Accepting AUC<.90<br>n=190	Accepting AUC≥.90<br>n=1,086	*p* [1]
Gender [%]	Female	51.4	50.7	53.1	.819	49.7	52.0	.583
	Male	48.6	49.3	46.9		50.3	48.0	
Age [M (SD)] in years		50.0 (18.2)	48.7 (18.3)	46.4 (18.0)	.030	48.2 (19.2)	49.0 (18.1)	.562
Household net income [M (SD)] in EUR		2,854 (1,642)	2,940 (1,730)	3,491 (2,159)	.001	2,848 (1,803)	3,059 (1,817)	.230
Education Diplomas/degrees from secondary/tertiary school [%]	Sec. Gen. School Leaving Cert.	24.2	20.6	13.9	<.001	23.2	19.9	<.001
	Intermediate School Degree	33.2	28.0	23.8		31.9	28.4	
	Leaving Cert. From Voc HS	6.9	9.2	6.8		7.1	7.9	
	College Entrance Exam	22.0	29.7	36.8		19.4	30.2	
	Other	9.4	8.2	17.6		9.2	11.0	
	Dropout, No School Certificate	2.9	2.3	0.0		6.1	1.2	
	Currently In School	1.4	2.0	1.1		3.0	1.3	
Vocational degree attained [%]	Apprenticeship	39.6	36.5	40.0	.126	36.1	39.1	.062
	Vocational School	10.7	9.0	5.8		7.8	9.1	
	Health Care School	0.0	0.0	0.0		0.0	0.0	
	Technical School	5.1	6.0	5.3		7.0	5.2	
	Civil Servant Training	1.7	2.7	2.1		1.4	2.3	
	Other Degree	1.8	1.7	0.8		2.4	1.4	
	Completed Voc. Training/Edu.	5.5	4.7	1.6		8.0	3.4	
	NA (e.g. college degree)	35.6	39.5	44.3		37.3	39.4	
Completed college education [%]	FH (Univ. of Applied Sciences)	4.9	8.0	7.2	.129	6.5	6.7	.217
	University, Technical College	13.1	9.3	14.4		7.5	12.6	
	College Not In Germany	0.1	0.3	0.0		0.8	0.1	
	Engineering, Technical School	1.5	1.9	0.2		0.9	1.4	
	University (East)	1.6	1.1	2.3		0.7	1.6	
	Graduation, state doctorate	2.8	3.3	4.3		2.5	3.5	

	Not applicable	76.0	76.1	71.6		81.0	74.1	
Occupation status [%]	Full time employment	37.4	42.5	43.3	.177	33.4	41.4	.108
	Part-time employment	13.8	11.1	14.9		12.1	13.2	
	Training/apprenticeship	3.2	3.5	1.0		5.2	2.4	
	Marginally employed	5.3	6.8	6.6		5.8	6.5	
	Partial retirement	0.0	0.5	0.0		0.0	0.2	
	Voluntary service	0.0	0.0	0.0		0.0	0.0	
	Workshop for the disabled	0.3	0.1	0.0		0.1	0.1	
	Not employed (anymore)	40.0	35.6	34.4		43.5	36.1	
Unemployment phases in past 10 years [%][2]	Yes	31.3	23.1	29.3	.050	38.0	25.7	.006
	No	68.7	76.9	70.7		62.0	74.3	
Body Mass Index (BMI) [M (SD)]		26.3 (5.0)	26.5 (7.6)	25.7 (4.9)	.226	25.4 (5.1)	26.4 (6.4)	.035
Self-reported health state [%][2]	Very good	9.0	12.8	18.2	.024	11.0	12.9	.042
	Good	39.8	41.4	39.7		34.8	41.2	
	Satisfactory	35.8	31.3	31.5		42.7	31.1	
	Not so good	12.4	11.9	9.8		9.8	12.2	
	Poor	3.0	2.7	0.9		1.7	2.6	
Health insurance status [%][2]	Statutory health insurance	90.0	87.6	86.4	.323	90.2	87.9	.465
	Private health insurance	10.0	12.4	13.6		9.8	12.1	
Risk preference [M (SD)] from 0 (avoidance) to 10 (preference)		4.6 (2.5)	4.4 (2.4)	4.4 (2.4)	.569	4.5 (2.6)	4.5 (2.4)	.962

1 Variance analyses and Chi^2-tests, respectively

2 Subsamples were presented with the items
